# Supplementary material for: Unique Metabolic Profiles of Korean Rice According to Polishing Degree, Variety, and Geo-Environmental Factors
Source: Foods. 2021 Mar 26;10(4):711. doi: 10.3390/foods10040711 (PMC8067222; doi:10.3390/foods10040711)
Supplement: Supplementary file 1 [file foods-10-00711-s001.pdf]

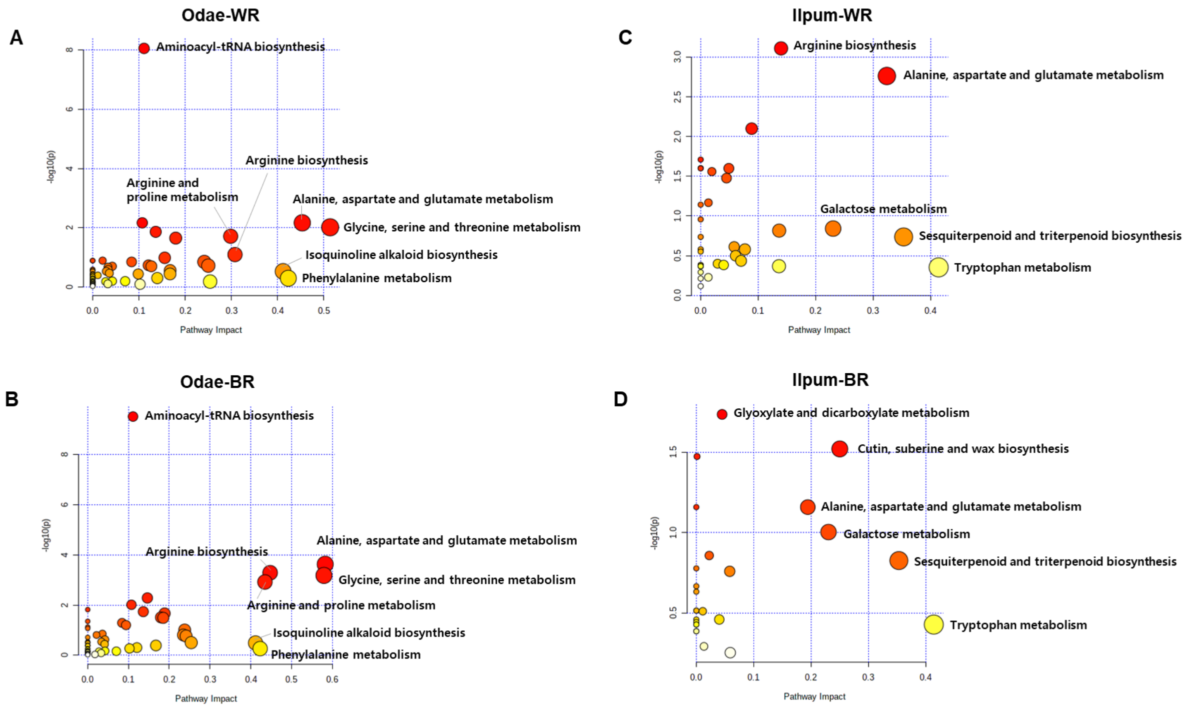

Figure S1. Pathway over-representation analysis of the metabolite profiles with significantly higher abundances in white (**A**) and brown rice (**B**) of Odae cultivar, and of Ilpum cultivar (C and D). The analysis is performed based on the hypergeometric test and relative-betweenness centrality in the *MetaboAnalyst* server (version 5.0).

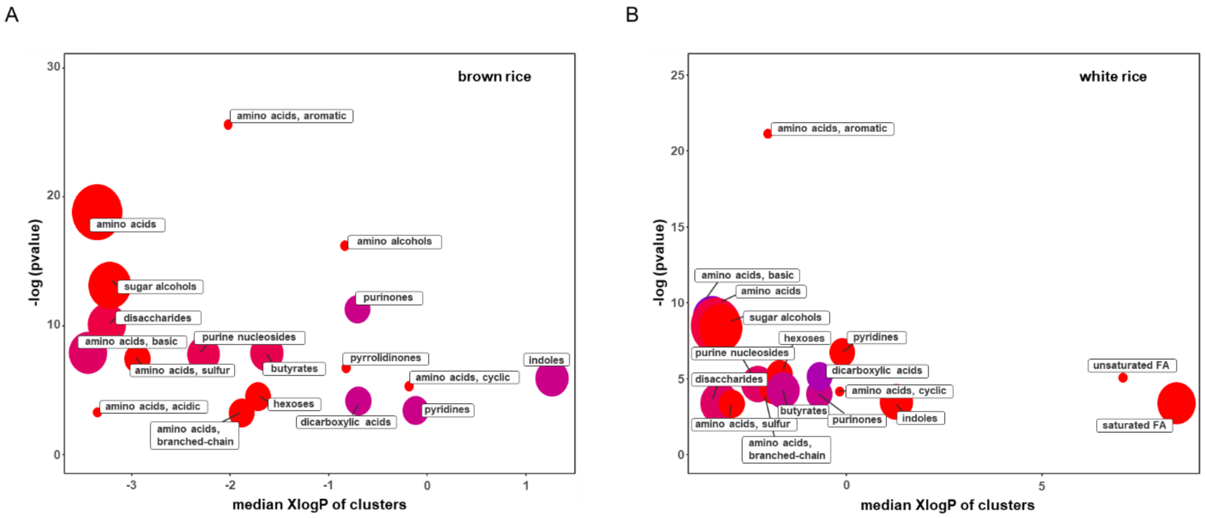

Figure S2. Chemical similarity enrichment analysis of cultivar-specific metabolites (Odae vs Ilpum) in brown rice (**A**) and white rice (**B**). Node size is proportional to the number of metabolites within each chemical class. Node color shows the ratio of metabolites with higher abundance and lower abundance. Red or blue colors indicate relatively higher ratio of higher abundance in Odae cultivar compared to Ilpum cultivar whereas purple color presents relatively equivalent ratio. *X*-axis and y-axis present partition coefficient and statistical significance (Kolmogorov–Smirnov test), respectively.

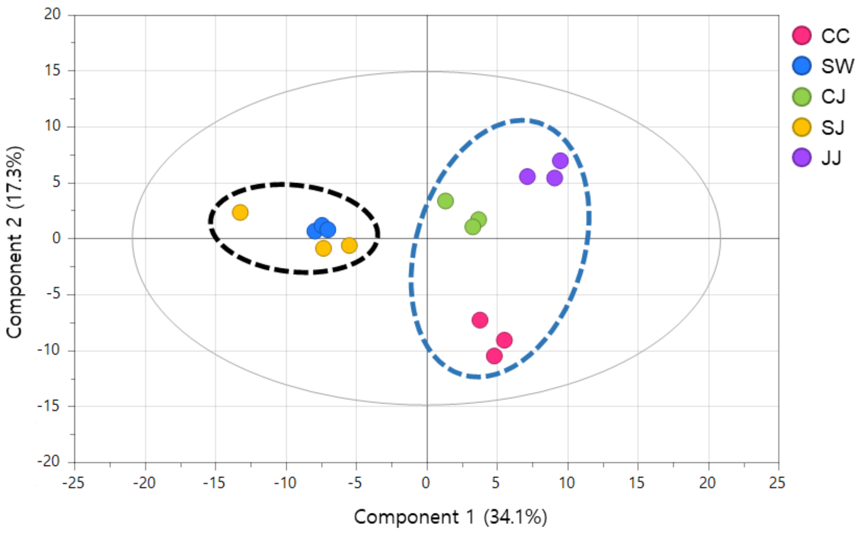

Figure S3. Metabolomic phenotype of brown rice of Odae cultivar. The score plot is generated based on principal component analysis (PCA). The variance is best explained by t1 vector (34.1%), which separates the profiles according to regions as follows: SW, SJ vs CC, CJ, JJ. CJ: Cheongju (Chungcheongbuk-do), SJ: Sangju (Gyeongsangbuk-do), SW: suwon (Gyeonggi-do), CC: Chuncheon (Gangwon-do), JJ: Jeonju (Jeollabuk-do)

Table S1. Cultivation regions of rice sample analyzed in the study.

Variety	Cultivation Region	
Odae/Ilpum	Gangwon-do	Chuncheon
	Gyeonggi-do	Suwon
	Chungcheongbuk-do	Cheongju
	Gyeongsangbuk-do	Sangju
	Jeollabuk-do	Jeonju
Total	5 regions and 2 cultivars	

Table S2. List of metabolites that are significantly different between the brown and white rice (fold-change is the ratio of brown rice to white rice). False discovery rate (FDR) was computed for adjusting multiple hypothesis testing by Benjamini-Hochberg.

Name	*P*-value	Fold change	FDR
9-cis-retinal	2.8E-08	5.79	6.5E-08
1-monopalmitin	1.5E-02	1.11	1.9E-02
1-monostearin	6.8E-03	1.10	8.7E-03
2,5-dihydroxypyrazine	9.7E-14	5.30	4.5E-13
2-deoxytetronic acid	3.8E-03	1.33	5.0E-03
2-hydroxycinnamic acid	2.2E-08	2.18	5.6E-08
3-methoxybenzaldehyde	1.5E-05	4.59	2.5E-05
4-guanidinobutyric acid	5.8E-13	3.49	2.5E-12
4-hydroxybenzaldehyde	4.2E-10	2.45	1.2E-09
4-hydroxycoumarin	5.4E-05	2.20	8.4E-05
4-methoxycinnamaldehyde	1.1E-04	3.47	1.7E-04
4-phenylbutyric acid	8.3E-03	2.76	1.1E-02
5-deoxy-5-methylthioadenosine	5.3E-04	1.49	7.3E-04
5-oxoete	2.3E-08	5.95	5.7E-08
8-hydroxyquinoline	1.3E-05	2.91	2.2E-05
9-oxo-10e,12e-octadecadienoic acid	1.1E-03	1.43	1.5E-03
9-oxo-ode	3.9E-08	2.37	8.7E-08
adenine	2.3E-02	1.58	2.8E-02
adenosine	8.1E-13	1.40	3.2E-12
adenosine-5-monophosphate	7.9E-19	4.60	1.0E-17
agmatine	9.8E-07	3.47	1.8E-06
alanine	7.8E-08	2.91	1.7E-07
allantoic acid dehydrated	4.9E-20	2.10	7.6E-19

arachidic acid	1.4E-10	1.67	4.3E-10
arginine	4.8E-13	2.73	2.1E-12
asparagine	6.3E-21	3.55	1.2E-19
asparagine dehydrated	1.2E-15	6.35	7.4E-15
aspartic acid	2.1E-22	4.79	5.5E-21
beta alanine	6.7E-12	2.73	2.3E-11
betaine	1.1E-11	2.01	3.7E-11
butane-2,3-diol	4.8E-02	1.92	5.8E-02
butyrolactam	6.2E-13	2.28	2.6E-12
cafestol	8.5E-08	12.35	1.8E-07
carnitine	1.7E-02	1.10	2.0E-02
cellobiose	2.9E-17	4.57	2.7E-16
cholesterol	1.2E-05	1.86	2.0E-05
choline	6.3E-11	2.75	2.0E-10
cis,cis-muconic acid	8.9E-03	1.30	1.1E-02
citric acid	6.4E-12	8.19	2.2E-11
citrulline	1.2E-17	3.38	1.2E-16
coumarin	8.3E-17	6.71	6.5E-16
cyanoalanine	1.2E-13	3.34	5.5E-13
cysteine	5.2E-12	4.73	1.9E-11
cystine	4.8E-02	1.24	5.7E-02
enolpyruvate	4.4E-18	1.68	4.9E-17
erythritol	5.8E-08	3.82	1.3E-07
fumaric acid	2.1E-22	3.32	5.5E-21
galactinol	1.5E-14	2.92	7.8E-14
gamma-aminobutyric acid	6.1E-08	2.99	1.3E-07
gluconic acid	2.9E-09	7.89	7.9E-09
glutamate	3.4E-20	4.73	5.9E-19
glutamine	9.0E-04	2.47	1.2E-03
glyceric acid	6.2E-07	2.07	1.2E-06
glycerol-alpha-phosphate	5.0E-32	3.69	3.9E-30
glycine	2.8E-06	2.16	4.9E-06
guanosine	3.4E-15	1.88	2.0E-14
heptadecanoic acid	3.4E-08	1.23	7.9E-08
hexitol	2.4E-08	2.02	5.9E-08
histidine	7.0E-06	2.91	1.2E-05
homoserine	1.3E-14	2.52	7.2E-14
hydroxylamine	7.7E-05	1.73	1.2E-04
hypoxanthine	2.5E-07	5.49	4.7E-07
inosine	1.9E-08	5.40	5.1E-08
inositol-4-monophosphate	9.6E-27	2.83	3.7E-25
isoleucine	1.2E-18	2.75	1.4E-17
isomaltose	3.2E-05	2.80	5.1E-05
leucine	2.0E-06	2.11	3.5E-06
glutathione reduced	1.9E-05	10.82	3.1E-05
lignoceric acid	5.9E-06	1.63	1.0E-05
lysine	1.1E-14	3.35	6.1E-14
lyxitol	1.0E-07	7.13	2.0E-07
lyxose	1.4E-04	1.46	2.1E-04
malic acid	1.0E-09	2.39	2.9E-09
maltol	8.2E-03	1.36	1.1E-02
maltotriose	4.4E-05	1.56	6.9E-05
melibiose	2.8E-14	3.30	1.4E-13
methionine	3.0E-10	6.69	9.3E-10
methionine sulfoxide	4.4E-03	1.47	5.7E-03
mono2-ethylhexyl phthalate mehp	5.7E-05	1.25	8.8E-05
monobutyl phthalate	2.5E-04	4.53	3.6E-04
myo-inositol	2.9E-32	3.05	3.9E-30
n3,n4-dimethyl-l-arginine	2.9E-14	2.80	1.4E-13
n6,n6,n6-trimethyl-l-lysine	2.2E-14	3.44	1.2E-13
n-acetyl-l-aspartic acid	1.0E-08	1.52	2.8E-08
n-acetylornithine	1.2E-04	1.70	1.8E-04

nicotinic acid	2.5E-07	2.11	4.8E-07
n-methylalanine	3.6E-08	2.33	8.1E-08
oleic acid	7.3E-13	1.52	2.9E-12
ornithine	7.5E-18	4.15	7.8E-17
oxalic acid	1.7E-07	1.94	3.5E-07
oxoproline	3.9E-19	3.77	5.5E-18
palmitic acid	5.2E-05	1.14	8.1E-05
phenylalanine	1.8E-07	2.31	3.5E-07
phloroglucinol	1.4E-09	2.17	3.9E-09
phosphoethanolamine	4.6E-16	4.74	3.1E-15
pipecolic acid	4.2E-10	2.48	1.2E-09
proline	2.5E-08	2.18	6.0E-08
prolylleucine	2.3E-16	2.94	1.7E-15
putrescine	6.0E-14	2.62	2.8E-13
pyruvic acid	4.4E-12	1.63	1.6E-11
raffinose	1.2E-15	3.06	7.4E-15
ribitol	7.5E-06	2.78	1.3E-05
serine	2.0E-12	3.00	7.6E-12
serotonin	3.4E-04	3.36	4.9E-04
sitosterol	9.7E-22	2.01	2.2E-20
skatole	2.7E-05	3.38	4.3E-05
sorbitol	2.2E-06	2.98	4.0E-06
squalene	1.9E-07	2.73	3.6E-07
stachydrine	2.1E-04	3.06	3.0E-04
succinic acid	1.8E-08	2.10	4.8E-08
thiamine	2.6E-08	1.72	6.1E-08
threonic acid	5.2E-12	3.54	1.9E-11
threonine	6.2E-17	3.03	5.1E-16
tocopherol alpha	3.2E-17	5.23	2.8E-16
trehalose	2.1E-09	6.69	5.8E-09
trigonelline	3.1E-04	2.84	4.5E-04
trisethylenglycol	6.3E-04	2.42	8.7E-04
tryptophan	1.0E-06	3.99	1.9E-06
tryptophan derivative	2.0E-05	3.27	3.2E-05
tyrosine	3.5E-10	2.79	1.1E-09
uric acid	3.5E-02	2.93	4.3E-02
uridine	6.1E-27	4.21	3.2E-25
valine	1.1E-16	2.63	7.8E-16
vanillin	6.7E-08	4.56	1.4E-07
xanthine	7.3E-04	1.99	9.9E-04
xylitol	2.4E-08	2.87	5.9E-08
xylose	9.6E-08	1.49	2.0E-07
2-hydroxypyridine	6.4E-04	0.55	8.7E-04
glycerophospho-n-palmitoyl ethanolamine	4.0E-04	0.75	5.6E-04
lactic acid	4.0E-03	0.81	5.3E-03

Table S3. List of metabolites that are significantly different between Odae and Ilpum cultivars in white rice (fold-change is the ratio of Odae to Ilpum). False discovery rate (FDR) was computed for adjusting multiple hypothesis testing by Benjamini-Hochberg.

Name	*P*-value	Fold change	FDR
trigonelline	3.8E-05	5.00	3.1E-04
tryptophan	5.2E-12	4.58	4.1E-10
butane-2,3-diol	1.9E-10	4.32	5.8E-09
skatole	3.3E-08	4.10	5.8E-07
tryptophan derivative	4.4E-08	3.83	6.8E-07
isomaltose	1.5E-03	3.48	6.2E-03
8-hydroxyquinoline	6.8E-08	2.78	8.9E-07
6-methylquinoline	4.0E-02	2.63	8.2E-02
inosine	6.8E-03	2.49	1.9E-02
serotonin	4.1E-02	2.40	8.3E-02

4-phenylbutyric acid	3.0E-03	2.28	1.0E-02
methionine sulfoxide	3.6E-05	2.13	3.1E-04
sphingosine	2.3E-05	2.02	2.1E-04
citric acid	2.4E-08	1.98	5.4E-07
xanthine	6.8E-05	1.97	4.6E-04
tyrosine	3.8E-06	1.83	4.2E-05
fructose	2.2E-03	1.83	8.5E-03
lignoceric acid	2.2E-10	1.79	5.8E-09
2,5-dihydroxypyrazine	5.3E-05	1.78	3.9E-04
nicotinic acid	2.8E-08	1.75	5.5E-07
myristic acid	1.8E-11	1.75	7.1E-10
2-hydroxypyridine	2.8E-03	1.74	1.0E-02
4-guanidinobutyric acid	4.7E-06	1.73	4.9E-05
2-amino-1,3,4-octadecanetriol	6.4E-05	1.72	4.6E-04
glucose	7.0E-04	1.71	3.3E-03
melibiose	7.7E-06	1.67	7.5E-05
phenylalanine	8.7E-04	1.63	4.0E-03
histidine	1.2E-04	1.63	7.6E-04
isoferulic acid	3.1E-04	1.59	1.7E-03
succinic acid	1.1E-03	1.59	4.7E-03
erythritol	2.5E-04	1.57	1.5E-03
alanine	1.1E-03	1.55	4.7E-03
serine	1.3E-03	1.52	5.6E-03
xylitol	4.1E-04	1.48	2.1E-03
cyanoalanine	2.8E-02	1.46	6.2E-02
glycine	2.6E-02	1.44	5.8E-02
sitosterol	1.6E-11	1.43	7.1E-10
proline	4.2E-03	1.42	1.3E-02
lyxitol	3.7E-03	1.42	1.2E-02
ribitol	5.9E-03	1.41	1.7E-02
methionine	3.7E-03	1.39	1.2E-02
adenosine	8.8E-08	1.39	1.1E-06
sorbitol	2.8E-03	1.34	1.0E-02
phloroglucinol	1.9E-02	1.33	4.3E-02
lysine	5.9E-04	1.32	2.9E-03
n-methylalanine	4.6E-03	1.32	1.3E-02
arachidic acid	1.4E-04	1.31	8.8E-04
valine	7.9E-03	1.30	2.1E-02
3-hydroxypyridine	2.9E-02	1.30	6.2E-02
gluconic acid	4.4E-03	1.30	1.3E-02
threonine	6.3E-03	1.28	1.8E-02
xylose	1.8E-02	1.27	4.1E-02
mannose	1.6E-02	1.27	3.8E-02
butyrolactam	2.4E-03	1.27	8.9E-03
putrescine	9.9E-03	1.26	2.5E-02
oleic acid	3.4E-03	1.24	1.1E-02
enolpyruvate	3.2E-03	1.20	1.1E-02
1-monopalmitin	9.2E-03	1.17	2.4E-02
carnitine	1.6E-02	1.14	3.9E-02
glutamine	3.8E-04	0.19	2.0E-03
raffinose	6.9E-08	0.39	8.9E-07
hydroxylamine	4.2E-03	0.44	1.3E-02
squalene	3.5E-13	0.45	5.5E-11
monobutyl phthalate	3.0E-02	0.49	6.4E-02
adenine	4.9E-02	0.50	9.6E-02
agmatine	1.8E-02	0.51	4.2E-02
uracil	1.3E-02	0.52	3.2E-02
galactinol	4.1E-05	0.52	3.2E-04
5-deoxy-5-methylthioadenosine	1.7E-03	0.56	6.9E-03
glyceric acid	3.9E-03	0.62	1.2E-02
n3,n4-dimethyl-l-arginine	3.1E-04	0.62	1.7E-03
4-hydroxycoumarin	1.3E-02	0.63	3.2E-02

oxalic acid	1.8E-03	0.63	7.0E-03
betaine	3.0E-04	0.64	1.7E-03
guanine	4.0E-02	0.68	8.2E-02
malic acid	2.5E-02	0.73	5.6E-02
homoserine	2.2E-02	0.73	4.9E-02
glycerophospho-n-palmitoyl ethanolamine	9.6E-03	0.78	2.5E-02
ornithine	4.3E-02	0.83	8.4E-02
cholesterol	3.6E-02	0.86	7.6E-02

Table S4. List of metabolites that are significantly different between Odae and Ilpum cultivar in brown rice (fold-change is the ratio of Odae to Ilpum).

Name	*P*-value	Fold change	FDR
trigonelline	6.8E-12	7.37	4.E-10
butane-2,3-diol	3.2E-04	6.96	1.E-03
l-glutathione (reduced)	3.0E-05	6.02	1.E-04
tryptophan	1.5E-10	4.41	5.E-09
skatole	3.7E-07	4.33	3.E-06
tryptophan derivative	2.5E-07	4.11	3.E-06
8-hydroxyquinoline	3.6E-07	3.53	3.E-06
serotonin	1.0E-03	3.30	3.E-03
citric acid	1.8E-11	2.94	7.E-10
xanthine	2.5E-05	2.91	1.E-04
isomaltose	9.1E-06	2.89	5.E-05
histidine	2.5E-05	2.89	1.E-04
methionine	1.8E-07	2.82	2.E-06
sorbitol	5.7E-05	2.64	2.E-04
nicotinic acid	7.5E-21	2.63	1.E-18
ribitol	3.8E-04	2.43	1.E-03
trehalose	1.7E-04	2.37	6.E-04
xylitol	1.5E-06	2.34	1.E-05
inosine	5.7E-04	2.26	2.E-03
2,5-dihydroxypyrazine	8.4E-10	2.24	2.E-08
tyrosine	7.4E-09	2.15	1.E-07
sphingosine	6.5E-06	2.13	4.E-05
lyxitol	9.7E-03	2.09	2.E-02
alanine	2.0E-04	2.09	7.E-04
cysteine	1.4E-05	2.03	7.E-05
2-amino-1,3,4-octadecanetriol	1.1E-04	1.98	4.E-04
n-methylalanine	2.1E-05	1.97	1.E-04
tocopherol alpha	3.3E-10	1.90	8.E-09
gamma-aminobutyric acid	1.4E-03	1.90	4.E-03
glycine	5.3E-04	1.89	2.E-03
erythritol	6.4E-03	1.89	1.E-02
phenylalanine	2.0E-04	1.87	7.E-04
succinic acid	4.8E-07	1.86	4.E-06
isoferulic acid	3.0E-05	1.82	1.E-04
serine	3.8E-06	1.79	2.E-05
beta alanine	2.8E-06	1.78	2.E-05
gluconic acid	2.3E-02	1.77	5.E-02
4-guanidinobutyric acid	3.7E-05	1.76	1.E-04
maltotriose	1.5E-05	1.75	8.E-05
agmatine	2.7E-02	1.73	5.E-02
proline	2.7E-04	1.71	9.E-04
melibiose	2.0E-05	1.67	1.E-04
lysine	4.3E-05	1.64	2.E-04
butyrolactam	4.7E-06	1.59	3.E-05
glucose	1.7E-03	1.58	5.E-03
pipecolic acid	2.7E-03	1.56	7.E-03
phosphoethanolamine	7.1E-04	1.56	2.E-03
fructose	2.9E-02	1.55	5.E-02

aspartic acid	6.0E-08	1.54	9.E-07
choline	4.5E-03	1.53	1.E-02
threonine	4.6E-05	1.50	2.E-04
valine	6.6E-06	1.48	4.E-05
glutamate	1.4E-04	1.47	5.E-04
putrescine	9.8E-04	1.46	3.E-03
phloroglucinol	5.2E-03	1.46	1.E-02
myristic acid	4.9E-10	1.46	1.E-08
2-deoxytetronic acid	1.2E-03	1.44	3.E-03
guanosine	4.9E-08	1.42	8.E-07
n6,n6,n6-trimethyl-l-lysine	1.2E-02	1.40	3.E-02
citrulline	1.8E-03	1.38	5.E-03
ornithine	6.2E-03	1.38	1.E-02
thiamine	3.3E-03	1.37	8.E-03
palmitoleic acid	2.9E-02	1.37	5.E-02
uridine	9.9E-07	1.35	8.E-06
cellobiose	1.8E-02	1.35	4.E-02
adenosine-5-monophosphate	1.0E-02	1.34	2.E-02
isoleucine	9.6E-04	1.33	3.E-03
sitosterol	1.5E-07	1.27	2.E-06
oxoproline	4.1E-02	1.24	7.E-02
arachidic acid	3.6E-02	1.21	7.E-02
pyruvic acid	2.3E-02	1.18	5.E-02
myo-inositol	2.3E-03	1.16	6.E-03
stearic acid	4.5E-02	1.08	8.E-02
squalene	5.9E-15	0.31	5.E-13
glutamine	3.1E-04	0.34	1.E-03
cafestol	1.4E-02	0.47	3.E-02
oxalic acid	9.8E-08	0.53	1.E-06
uracil	1.2E-02	0.55	3.E-02
galactinol	1.5E-06	0.62	1.E-05
9-oxo-ode	1.0E-02	0.64	2.E-02
glyceric acid	6.4E-03	0.65	1.E-02
raffinose	1.6E-06	0.65	1.E-05
guanine	2.7E-02	0.68	5.E-02
lactic acid	3.6E-04	0.72	1.E-03
glycerophospho-n-palmitoyl ethanolamine	2.4E-02	0.77	5.E-02
2,3-dihydroxypyridine	1.0E-02	0.83	2.E-02
mono(2-ethylhexyl) phthalate (MEHP)	4.6E-02	0.84	8.E-02
adenosine	1.3E-06	0.88	1.E-05
indirubin	4.8E-02	0.93	8.E-02
